# Ciprofol versus propofol for sedation in colonoscopy: a systematic review and meta-analysis of randomized controlled trials

**DOI:** 10.1016/j.bjane.2025.844710

**Published:** 2025-11-17

**Authors:** Saul Dominici, Italo C. Martins, Breno Dias L. Ribeiro, Victor Arthur Ohannesian, Brunno Braga Sauaia, Abdias Rocha Santos, Caio Márcio Barros de Oliveira, Plínio da Cunha Leal

**Affiliations:** a Universidade Federal do Maranhão, Departamento de Medicina, São Luís, MA, Brazil; b Faculdade de Ciências da Saúde Albert Einstein de Israel (FICSAE), Departamento de Medicina, São Paulo, Brazil; c Hospital Djalma Marques, Departamento de Medicina, São Luís, MA, Brazil

**Keywords:** Anesthetics, Colonoscopy, Meta-analysis, Propofol, Safety, Treatment outcome

## Abstract

**Background:**

Ciprofol has emerged as a potential alternative sedative with improved safety and efficacy. However, comparative data for colonoscopy sedation remain limited.

**Methods:**

A systematic search in PubMed, Embase, Cochrane Library, and Web of Science identified RCTs published through August 2025. Studies included patients undergoing colonoscopy using ciprofol or propofol, reporting relevant efficacy or safety outcomes. Risk Ratios (RRs) and Mean Differences (MDs) were calculated using the Mantel-Haenszel random-effects model and 95% Confidence Intervals. The heterogeneity was assessed with *I*² statistics and Cochrane Q test. Primary outcomes were procedure success rate and patient satisfaction (assessed on a 1-to-10 scale). Secondary outcomes included sedation onset time(s), respiratory depression, injection pain, and hemodynamic adverse events (hypotension and bradycardia). The statistical analyses were performed in *R* software (version 4.4.1.)

**Results:**

Three RCTs with 645 patients were included. Colonoscopy success rates were similar between ciprofol and propofol (RR = 1.005; 95% CI 0.992–1.019). Ciprofol showed a lower risk of respiratory depression (RR = 0.24; 95% CI 0.08–0.71), injection pain (RR = 0.04; 95% CI 0.01–0.15), and hypotension (RR = 0.85; 95% CI 0.75–0.96). Patient satisfaction was slightly higher with ciprofol (MD = 0.18; 95% CI 0.08–0.29). No significant differences were found in sedation onset time (s) (MD = 2.49s; 95% CI -3.77–8.74) or bradycardia (RR = 0.88; 95% CI 0.44–1.77).

**Conclusion:**

Ciprofol provides comparable efficacy to propofol for colonoscopy sedation, with a lower incidence of respiratory depression, injection pain, and hypotension. Patient satisfaction was slightly higher with ciprofol, while bradycardia occurrence was similar. These findings suggest ciprofol as a promising alternative, though further large-scale studies are needed to confirm its clinical benefits.

## Introduction

Colonoscopy is a cornerstone endoscopic procedure for the diagnosis, prevention, and treatment of colonic diseases, playing a pivotal role in the early detection of colorectal cancer ‒ one of the leading causes of mortality worldwide.^1^ Effective sedation is essential in this context, not only to ensure patient comfort but also to optimize procedural conditions, enhancing the quality and safety of the examination.^2^ Propofol has long been the sedative agent of choice for endoscopic procedures, primarily due to its rapid onset of action, short duration, and favorable recovery profile, which are particularly advantageous in ambulatory settings.^3^ However, its use is not without challenges, as it is associated with risks such as respiratory depression and hemodynamic instability, necessitating close monitoring and dosage adjustments to mitigate adverse effects.^4^^,^^5^

In the search for alternatives that combine efficacy with a potentially improved safety profile, ciprofol has emerged as a promising structural analog of propofol. Early studies suggest that ciprofol offers comparable ‒ if not superior ‒ sedative efficacy, with a reduced incidence of adverse events and faster recovery times.^6^ Nevertheless, the variability in findings across clinical trials highlights the need for a robust quantitative analysis to consolidate the evidence and provide a nuanced understanding of the relative benefits and risks of each agent in the context of colonoscopy. Despite the widespread use of sedation in colonoscopies, the literature lacks a comprehensive comparative evaluation of the efficacy and safety profiles of ciprofol and propofol. While previous meta-analyses have compared ciprofol and propofol, their scope has been substantially broader, thereby limiting their applicability to this specific procedural context. For instance, existing reviews have aggregated data from diverse surgical and non-surgical procedures or focused on the induction and maintenance of general anesthesia rather than procedural sedation.^7^^,^^8^ Consequently, a critical knowledge gap persists regarding the relative merits of these agents specifically for colonoscopy, a procedure with unique physiological demands and patient safety considerations.

Therefore, this meta-analysis aims to systematically assess and compare ciprofol and propofol in colonoscopy procedures, focusing on critical outcomes such as adverse event rates and recovery metrics. The findings will provide a comprehensive synthesis of the available evidence, offering valuable insights for clinical practice and guiding future research.

## Methods

This systematic review and meta-analysis of the literature was performed and reported following the Cochrane Collaboration Handbook for Systematic Review of Interventions and the Preferred Reporting Items for Systematic Reviews and Meta-analysis (PRISMA) Statement guidelines.^9^^,^^10^ The review protocol was prospectively registered on International Prospective Register of Systematic Reviews (PROSPERO) in November 2024 under protocol CRD42024613088.

### Eligibility criteria

Original studies were included in this review based on the following eligibility criteria: 1) Randomized Controlled Trial reports published in peer-reviewed journals; 2) Patients undergoing Colonoscopy; 3) Ciprofol and propofol comparison groups; 4) At least one safety or efficacy endpoint of interest. Non-randomized observational studies, non-English reports, literature reviews and conference abstracts were excluded from this study. No restrictions were applied regarding minimum sample size, patient age, or intervention dose range, provided that the studies fulfilled the inclusion criteria of randomized controlled design, colonoscopy patients, and a direct comparison between ciprofol and propofol. We acknowledge that the restriction to English-language reports, although commonly applied in systematic reviews, may have introduced language bias; however, this choice was made to ensure uniformity in data extraction and to minimize the risk of misinterpretation during the analysis.

### Search strategy and data extraction

A systematic search was performed in PubMed, Embase, Cochrane Central Register of Controlled Trials and Web of Science databases from inception to August 2025 using the following search-key strategy: (Ciprofol OR HSK3486 OR Propofol) AND (Colonoscopy OR "Colonoscopy Procedure" OR "Colonoscopy Surgery" OR Colonoscop* OR Colonoscopic) AND (Sedation OR Anesthesia OR Anaesthesia OR Analgesia OR "Conscious Sedation" OR "Moderate Sedation" OR "Procedural Sedation"). Two independent authors (S.D and I.C.M) screened titles and abstracts for eligibility evaluation.

The included articles’ data were independently extracted by two authors (S.D. and B.B.S) who reviewed the reports, supplementary materials and extracted the RCTs’ characteristics and relevant information. The discrepancies were discussed and settled by another two authors (I.C.M and V.A.O)

### Endpoints

The efficacy endpoints of this meta-analysis were (1) Patient satisfaction and (2) Onset time of sedation (s) and (3) Success rate of colonoscopy, and the safety outcomes were (4) Bradycardia, (5) Hypotension, (6) Injection pain, (7) Respiratory depression. Patient Satisfaction was measured by different surveys on a scale of 1 to 10.

### Quality assessment

The risk of bias in the included studies was assessed using the Revised Cochrane Risk of Bias Tool for Randomized Trials (RoB-2). Two independent reviewers (V.A.O and I.C.M) conducted the evaluation based on the guidelines outlined in the Cochrane Handbook for Systematic Reviews of Interventions.^9^^,^^11^ Any disagreements were addressed through discussion, and if a consensus was not reached, a third reviewer (S.D) was consulted for resolution.

The assessment covered various domains, including random sequence generation, allocation concealment, blinding of participants and study personnel, blinding of outcome assessors, completeness of outcome data, selective reporting of results, and additional potential sources of bias. Each domain was judged as “low risk of bias”, “some concerns”, or “high risk of bias”, following the standardized RoB2 algorithm. To ensure transparency, domain-level judgments were combined to produce an overall risk of bias rating for each included trial. A study was classified as “low risk of bias” if all domains were rated as low risk, “high risk of bias” if at least one domain was judged high risk, and “some concerns” if one or more domains raised concerns without being rated high risk. The results of the risk of bias assessment were summarized in graphical format to facilitate interpretation and reproducibility.

### Statistical analysis

We analyzed the endpoints using Risk Ratio (RR) for binary outcomes and Mean Difference (MD) for continuous outcomes, presenting them with 95% Confidence Intervals (95% CI). The Mantel-Haenszel (MH) method was applied with a random-effects model. To assess heterogeneity, we utilized the *I*^2^ statistic and the Cochran *Q* test, considering heterogeneity significant when *I*^2^ exceeded 40% and p-values were below 0.1.^9^ We chose a more liberal p-value threshold (p < 0.1) for the Cochran *Q* test to enhance its sensitivity in detecting heterogeneity, particularly in meta-analyses with a small number of included studies, as this test is known to have low statistical power under these conditions. This approach helps to avoid Type II errors (falsely concluding homogeneity). For outcomes exhibiting significant heterogeneity (*I*^2^ ≥ 40%), a leave-one-out sensitivity analysis was conducted to determine whether any single study disproportionately influenced the results. This approach also helped identify studies contributing most to the overall heterogeneity of those outcomes. All statistical analyses were performed using *R* software (version 4.4.1, R Foundation for Statistical Computing, Vienna, Austria).^12^

## Results

### Study selection

**Figure 1:**
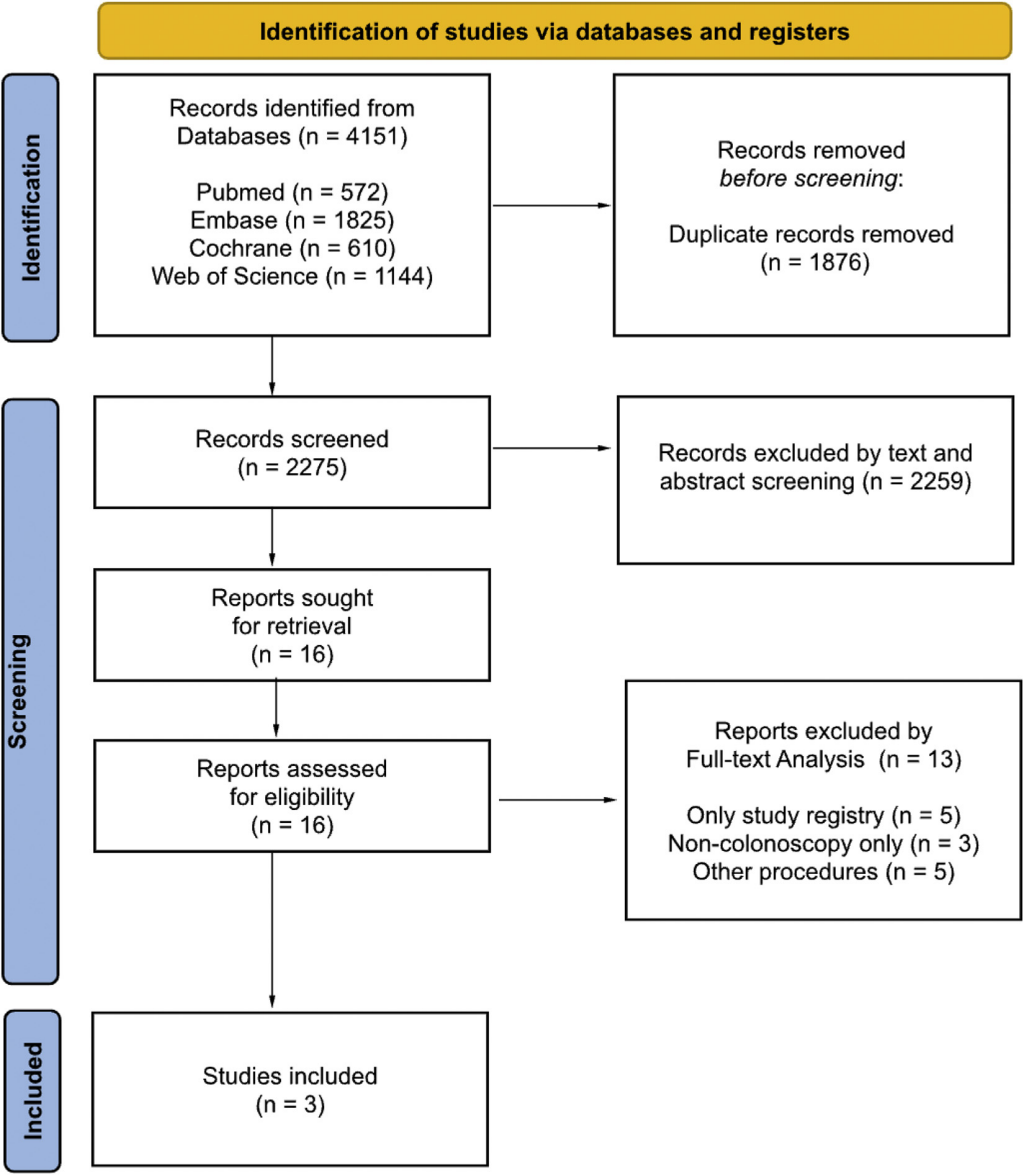
The prisma flow diagram.

The initial search identified 4151 articles, with 572 from PubMed, 1825 from Embase, 1144 from Web of Science and 610 form Cochrane Central Register of Controlled Trials. After the removal of 1876 duplicates, 2275 articles underwent title and abstract screening. Of these, 16 were deemed eligible for full-text review. Among them, 13 were later excluded, with the reasons for exclusion detailed in the Prisma Flow Diagram, shown in Figure 1. Ultimately, a total of 3 RCTs were included.^13^, ^14^, ^15^

### Characteristics of the included studies and patients

A total of three studies, RCTs conducted exclusively in China, including 645 patients (44% male) were analyzed.^13^, ^14^, ^15^ Their clinical baseline features are shown in Table 1.

**Table 1:** Baseline characteristics of the included studies.

Baseline clinical features	Li (2022)		He (2024)		Gao (2024)	
	Ciprofol	Propofol	Ciprofol	Propofol	Ciprofol	Propofol
Patients (n)	129	130	110	112	82	82
Age (years)	43.8 ± 11.8	44.1 ± 11.3	48.0 ± 11.2	49.0 ± 9.7	54 ± 15.56	54 ± 14.07
Gender						
Male (%)	55 (38.2)	63 (43.4)	54 (49.1)	46 (41.1)	34 (41.5)	32 (40)
Female (%)	89 (61.8)	82 (56.6)	56 (50.9)	66 (58.9)	48 (58.5)	50 (40)
Height (mean ± SD, cm)	161.5 ± 8.2	163.1 ± 8.4	166.2 ± 9.0	165.2 ± 7.5	166 ± 8,89	165.5 ± 8.15
Weight (mean ± SD, kg)	60.0 ± 9.6	61.5 ± 9.7	65.9 ± 12.0	65.1 ± 10.3	63.5 ± 12.59	63.5 ± 12.59
BMI (mean ± SD, kg m^-2^)	23.2 ± 2.5	23.4 ± 2.6	23.7 ± 2.9	23.8 ± 2.7	23.4 ± 3.0	23.7 ± 3.0
ASA PS						
I (%)	115 (79.9)	118 (81.4)	29 (26.4)	36 (32.1)	16 (19.5)	20 (24.4)
II (%)	29 (20.1)	27 (18.6)	81 (73.6)	76 (67.9)	66 (80.5)	62 (85.6)

Objectives	Compare the deep sedation properties of ciprofol and propofol using an 8% non-inferiority margin in patients undergoing gastroscopy and colonoscopy.		Evaluate whether ciprofol provides greater hemodynamic stability than propofol during colonoscopy.		Assess differences in safety and efficacy between ciprofol and propofol for painless colonoscopy.	

ASA PS, American Society of Anesthesiologists Physical Status; BMI, Body Mass Index; SD, Standard Deviation.

The three studies have similar inclusion criteria, with slight variations regarding age range, clinical parameters, and specific exclusion conditions. In Gao et al. (2024), patients aged ≥ 18 years, with an ASA physical status I–II and BMI between 18 and 30 kg.m^-2^, were included. He (2024) adopted similar criteria but restricted the age range to 18–65 years and added a painless colonoscopy duration of < 20 minutes as an inclusion criterion. Li (2022) included patients aged ≥ 18 and < 65 years with a BMI between 18 and 30 kg.m^-2^.^13^, ^14^, ^15^

Regarding exclusion criteria, Gao et al. (2024) and He et al. (2024) share criteria such as BMI ≥ 30 kg.m^-2^, a history of substance abuse, allergies to anesthetics, and pregnancy or lactation. On the other hand, Li et al. (2022) presents stricter exclusion criteria, including contraindications for general and deep sedation, allergy to soybean- or egg-based products, recent use of propofol, benzodiazepines, opioids, or any analgesic-containing formulation within the past 72 hours. Additionally, Li (2022) excluded patients with neutropenia, thrombocytopenia, hepatic dysfunction, and renal insufficiency. Patients with uncontrolled hypertension (SBP ≥ 170 mmHg, DBP ≥ 105 mmHg), severe arrhythmias, heart failure, unstable angina, recent myocardial infarction, and advanced atrioventricular blocks were also excluded. He et al. (2024) further added arrhythmia and participation in pharmacological clinical trials within the last 3 months as exclusion criteria.^13^, ^14^, ^15^

### Technical aspects of the colonoscopy procedure

Regarding monitoring, all studies assessed vital parameters such as blood pressure, heart rate, oxygen saturation, and ECG during the procedure. However, He et al. (2024) monitored Heart Rate Variability (HRV) and recorded data every 2 minutes for 20 minutes after induction. Oxygen supplementation also varied among the studies: Gao (2024) and He (2024) used a nasal cannula at 4 L.min^-1^ and 5 L.min^-1^, respectively, while Li et al. (2022) administered oxygen at 10 L.min^-1^ via a face mask until the patient fully regained consciousness. Regarding pre-procedure preparation, all patients underwent standardized bowel preparation.^13^, ^14^, ^15^

For anesthetic induction, some studies administered fentanyl before ciprofol or propofol, but with differences in dosage and reinforcement regimens. He (2024) used 0.05 μg.kg^-1^ of sufentanil, whereas Li et al. (2022) administered 50 μg of fentanyl. Induction was performed with ciprofol (0.4 mg.kg^-1^) or propofol (2.0 mg.kg^-1^) in Gao et al. (2024) and He et al. (2024), but in Li et al. (2022), the propofol dose was lower (1.5 mg.kg^-1^). The criterion for initiating colonoscopy was similar across all three studies, requiring a MOAA/S score of ≤ 1, assessed every 30 seconds during induction. However, the frequency of monitoring during the maintenance phase varied, occurring every 5 minutes in Gao et al. (2024) and every 2 minutes in Li et al. (2022) and He et al. (2024).^13^, ^14^, ^15^

During the sedation maintenance phase, the additional dosing regimen differed among the studies. Gao et al. (2024) and Li et al. (2022) administered reinforcement doses of 0.1 mg.kg^-1^ for ciprofol and 0.5 mg.kg^-1^ for propofol, while He et al. (2024) used supplementary doses equivalent to one-third of the initial dose. Sedation was considered ineffective if more than five additional doses were required within 15 minutes in all studies, in which case propofol was the only permitted alternative sedative.^13^, ^14^, ^15^

In the postoperative period, all patients were transferred to the Post-Anesthesia Care Unit (PACU), and discharge was based on standardized scoring systems. Gao et al. (2024) used the Post Anesthesia Discharge Scoring System (PADSS) with a discharge threshold of ≥ 9, while He (2024) and Li (2022) used the modified Aldrete score, with a discharge criterion of ≥ 9.^13^, ^14^, ^15^

### Pooled analysis of included studies

#### Success rate of colonoscopy

**Figure 2:**
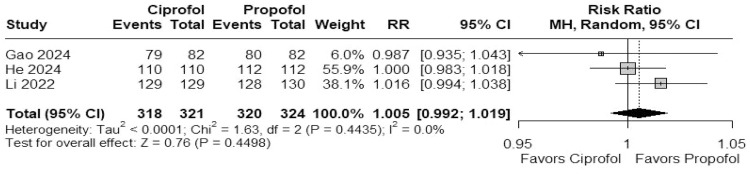
Forest plot showing no significant difference in success rate of colonoscopy between ciprofol and propofol.

In a meta-analysis of 3 studies, no significant difference was observed in the success rate of colonoscopy between ciprofol and propofol (RR = 1.005; 95% CI 0.992–1.019; *I*^2^ = 0%; p = 0.4498; Fig. 2).

#### Onset time to sedation(s)

**Figure 3:**
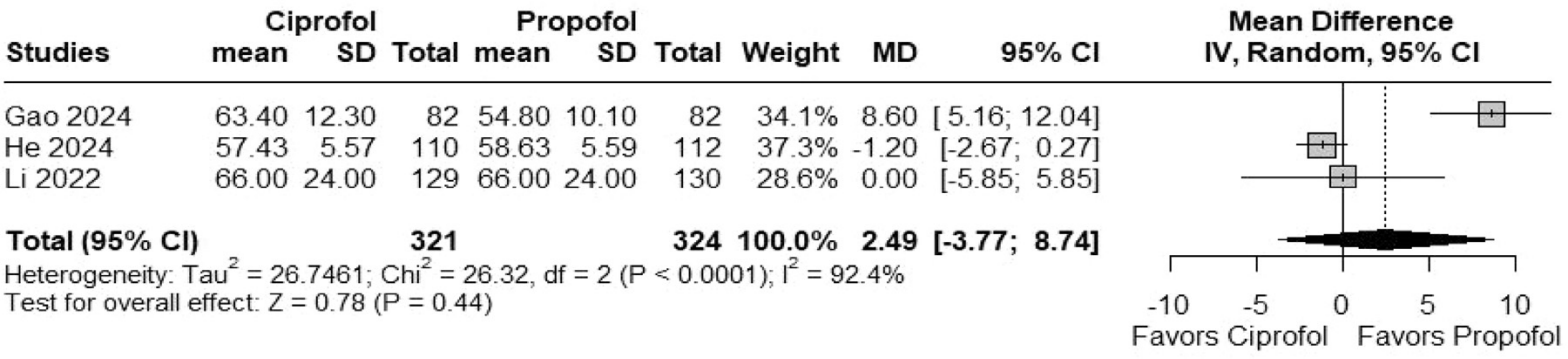
Forest plot showing no significant difference in onset time to sedation between ciprofol and propofol.

In a meta-analysis of 3 studies, the time to onset of sedation showed no statistically significant difference between ciprofol and propofol (MD = 2.49; 95% CI -3.77–8.74; *I*^2^ = 92.4%; p = 0.4356; Fig. 3).

#### Respiratory depression

**Figure 4:**
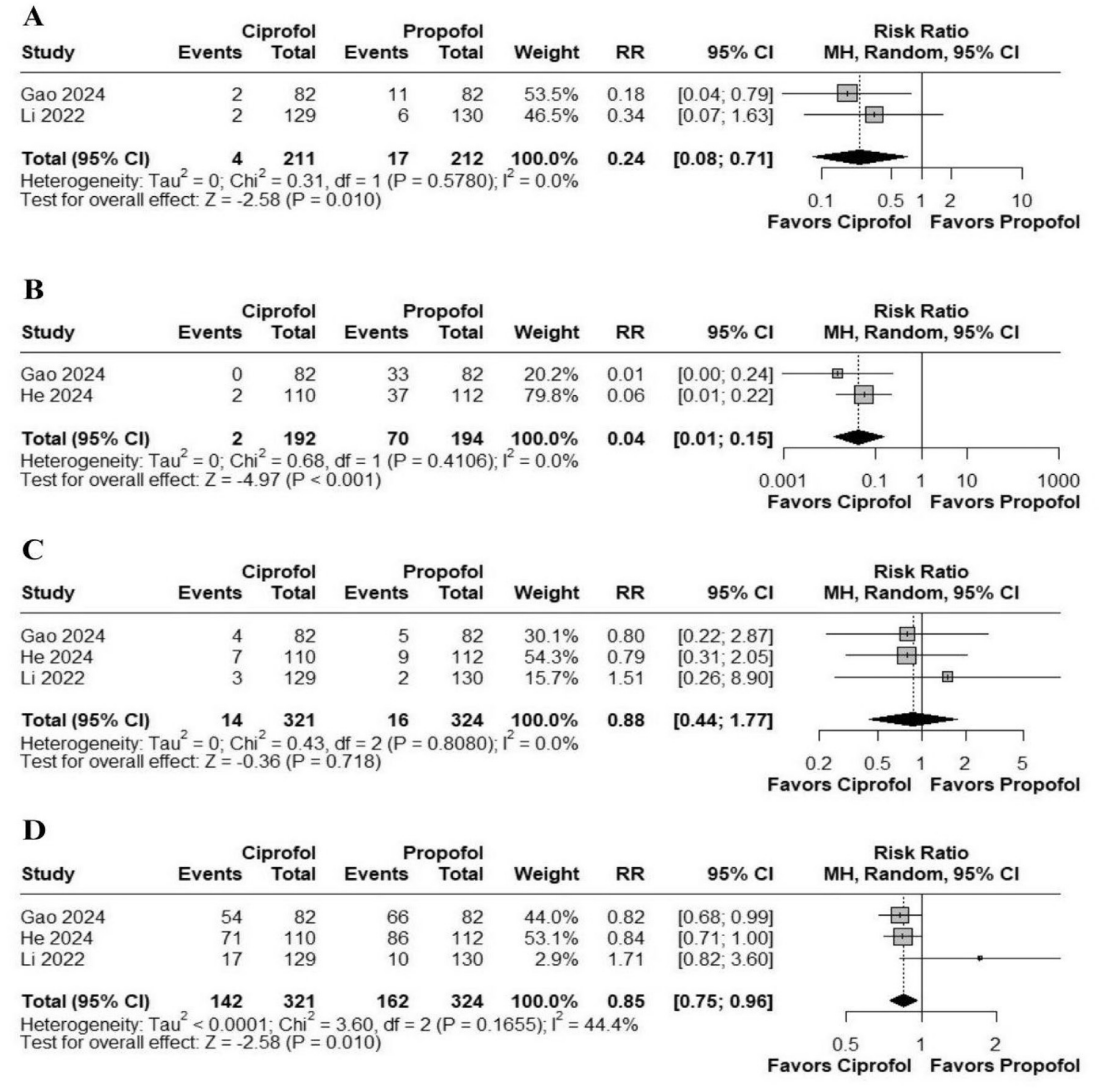
Forest plots of safety outcomes comparing ciprofol and propofol. Panel (A) shows the risk ratio for respiratory depression. Panel (B) shows the risk ratio for injection pain. Panel (C) shows the risk ratio for bradycardia. Panel (D) shows the risk ratio for hypotension.

In a meta-analysis of 3 studies, ciprofol was associated with a significantly lower risk of respiratory depression compared to propofol (RR = 0.24; 95% CI 0.08–0.71; *I*^2^ = 0%; p = 0.01; Fig. 4).

#### Injection pain

In a meta-analysis of 2 studies, ciprofol significantly reduced the occurrence of injection pain compared to propofol (RR = 0.04; 95% CI 0.01–0.15; *I*^2^ = 0%; p < 0.001; Fig. 4).

#### Hypotension

In a meta-analysis of 3 studies, ciprofol was associated with a lower risk of hypotension compared to propofol (RR = 0.85; 95% CI 0.75–0.96; *I*^2^ = 44.4%; p = 0.010; Fig. 4).

#### Bradycardia

In a meta-analysis of 3 studies, no significant difference was observed in the incidence of bradycardia between ciprofol and propofol (RR = 0.88; 95% CI 0.44–1.77; *I*^2^ = 0%; p = 0.718; Fig. 4).

#### Patient satisfaction

**Figure 5:**
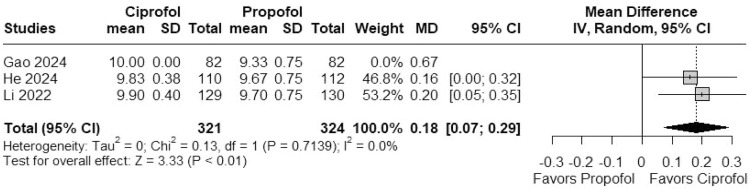
Forest plot showing a significant rise in patients’ satisfaction between ciprofol and propofol.

In a meta-analysis of 3 studies, ciprofol was associated with significantly higher patient satisfaction compared to propofol (MD = 0.18; 95% CI 0.07–0.29; *I*^2^ = 0%; p < 0.01; Fig. 5).

### Sensitivity analysis

To assess heterogeneity, a leave-one-out analysis was performed. For onset time to sedation(s), Li et al. (2022) was the primary contributor to heterogeneity. Its exclusion reduced *I*^2^ to 0% and yielded MD = -1.13 (95% CI: -2.55 to 0.30; p = 0.1202), showing no significant difference. Omitting He et al. (2024) (MD = 4.64; *I*^2^ = 83.8%) and Gao et al. (2024) (MD = 3.57; *I*^2^ = 96.2%) did not resolve heterogeneity. The overall random-effects model showed MD = 2.49 (95% CI: -3.77 to 8.74; p = 0.4356) with high heterogeneity (I^2^ = 92.4%; Supplementary Material 1).

For hypotension, removing Li et al. (2022) reduced *I*^2^ to 0% (RR = 0.83 [0.73–0.94]), while excluding Gao et al. (2024) (RR = 1.09; *I*^2^ = 70.2%) and He et al. (2024) (RR = 1.08; *I*^2^ = 72.0%) maintained moderate heterogeneity. The random-effects model showed RR = 0.85 (95% CI: 0.75–0.96) with *I*^2^ = 44.4%, favoring ciprofol over propofol in reducing hypotension risk (Supplementary Material 2).

### Risk of bias of included studies

All three included RCTs were considered to have a low risk of bias across all assessed domains.^13^, ^14^, ^15^ The three (3/3; 100%) trials demonstrated low risk in relation to the randomization process, deviations from intended intervention, missing outcome data, measurement of outcomes, or selection of reported results. Detailed results of the RoB-2 assessment are provided in Supplementary Material 3.

## Discussion

Clinical characteristics and outcomes of the novel compound known as ciprofol have been recently researched and compared with propofol in different medical procedures. This meta-analysis of three RCTs encompassing 645 patients was the first to compare the safety and efficacy between these two short-acting intravenous anesthetics in patients undergoing colonoscopy procedure. The results we found suggest that ciprofol administration was less likely to cause respiratory depression and hypotension than propofol. Patients undergoing colonoscopy with ciprofol had less injection pain and higher levels of procedure satisfaction when compared to those under the propofol effect. However, the procedure success rate, onset time to sedation(s) and bradycardia were not significantly influenced by the anesthetic compound choice. Clinically, this suggests that while efficacy remains similar, the improved safety profile of ciprofol could reduce the burden of managing hemodynamic instability during procedures, potentially lowering the need for immediate interventions such as vasopressors or supplemental oxygen.

Colonoscopy is a vital procedure for diagnosing and preventing colorectal diseases, enabling early detection and intervention for conditions like cancer and polyps. Sedation and analgesia are important in this exam but the pattern protocol for this procedure anesthesia is not well established, with hospitals adopting their own protocols according to their clinical experience and structure. Still, the use of benzodiazepines and opioids (alone or combined) are the most common pharmacological tools used in colonoscopy.^2^^,^^5^^,^^16^, ^17^, ^18^, ^19^ Propofol, the “Milk of Amnesia” used to maintain general anesthesia and/or sedation in invasive and non-invasive medical procedures, has been widely used in gastroscopy due to its rapid anesthetic effect and fast patient recovery, reducing long-time sedation adverse effects.^20^ However, propofol administrations can cause hemodynamic adverse effects, like hypotension, bradycardia, respiratory depression and the rare and lethal Propofol-Related Infusion Syndrome (cardiovascular affections, metabolic acidosis, lactic acidosis, rhabdomyolysis, hyperkalemia, lipidemia, hepatomegaly and acute renal failure).^21^, ^22^, ^23^

Ciprofol (HSK3486), a novel-short acting intravenous anesthetic based on a propofol structural modification was developed and reported in China in 2017, aiming to improve efficacy and reduce adverse effects.^6^ It presents the chemical structure “(R)-2-(1-cyclopropyl ethyl)-6-isopropylphenol” and acts as a gamma-aminobutyric acid type A (GABA_A_) antagonist and positive allosteric modulator with higher potency and selectivity than propofol, allowing a lower dose pharmacological administration.^6^^,^^24^ This molecule presents higher plasma protein binding, and faster distribution with hepatic metabolism via CYP2B6 and CYP2C19 followed by primary renal excretion, demonstrating a lower systemic accumulation, faster half-life elimination and consequently a rapid recovery.^6^^,^^24^^,^^25^ This anesthetic has been recently tested alone or compared with propofol in RCTs, cohort studies and case reports from diverse medical areas with invasive and non-invasive procedures.^15^^,^^26^, ^27^, ^28^, ^29^, ^30^, ^31^, ^32^

In this study, no significant statistical difference was evidenced between propofol and ciprofol for the success rate of colonoscopy and the time from drug administration to sedation. Our findings about the procedure success rate are consistent with a meta-analysis of 19 RCTs with diverse surgical and non-surgical procedures by Saeed et al. (2024) which presented that ciprofol has an efficacy comparable to propofol for endoscopic procedure completion rate and anesthesia/sedation induction time.^7^ However, a meta-analysis of six RCTs regarding the use of these two drugs for induction and maintenance of general anesthesia by Hudaib et al. (2024) reviewed the time to successful induction and highlighted a propofol advantage in comparison with the ciprofol dosage of 0.5 mg.kg^-1^ and no advantage on 0.4 mg.kg^-1^.^8^ Ultimately, in a non-randomized phase II trial the colonoscopy was 100% successful in the ciprofol and propofol groups.^33^ The literature and our results suggest that ciprofol is an effective compound like propofol for colonospic procedures and this might be explained because of their structure similarity.

Although our results did not show a statistically significant difference in sedation onset time, the high heterogeneity (*I*^2^ = 92.4%) identified for this outcome requires a more detailed analysis. Our leave-one-out sensitivity analysis demonstrated that the study by Li et al. (2022) was the primary contributor to this heterogeneity, as its exclusion reduced the *I*^2^ to 0%.^15^ A deeper evaluation of the included studies' characteristics reveals that methodological differences may be the cause. Specifically, the study by Li et al. (2022) used a lower propofol dose (1.5 mg.kg^-1^) compared to the other studies (2.0 mg.kg^-1^).^15^ Furthermore, while Li et al. (2022) administered fentanyl as premedication, He et al. (2024) used sufentanil, which may have influenced the onset of sedation.^14^^,^^15^ These variations in dosage and anesthetic technique, along with stricter patient exclusion criteria in the Li et al. (2022) study, may have impacted the results, making it an outlier in our analysis.^15^ This similarity allows both drugs to act as positive allosteric modulators of GABA_A_ receptors, leading to comparable sedative and anesthetic effects, including similar induction times and procedural success rates.^6^

Regarding ciprofol safety, our findings suggest that this drug is less likely to cause respiratory depression and hypotension than propofol, but bradycardia during the novel drug effect had no significant difference compared to the control group. In a meta-analysis of 7 RCTs, Zeng et al. (2024) demonstrated that patients undergoing surgery or painless examination under ciprofol administration had a significantly lower incidence of respiratory depression, consenting with the results of our study. Meanwhile, the incidence of hypotension was not statistically relevant compared to the patients under propofol.^34^ Therefore, a prospective single-center cohort encompassing 200 patients undergoing painless colonoscopy registered 3 cases of respiratory depression, 2 occurrences of bradycardia and 76 hypotension registrations, representing that 38% of the sample had hemodynamic adverse events due to blood pressure.^35^ In total, the cases registered of hypotension in our analysis have an approximate incidence of 44.2% in the ciprofol sample, but still less than 50% in the propofol population we included. From a clinical perspective, these safety advantages may translate into reduced requirements for intensive cardiopulmonary monitoring, shorter recovery room stays, and improved workflow efficiency in busy endoscopy centers. The respiratory depression reduction and lower incidence of hypotension in ciprofol administration might be explained by its higher potency and more stable plasma profile, leading to lower dose administration of this drug and consequently, a gentler modulating of GABA_A_ receptors, preserving the respiratory drive and the hemodynamic stability.^24^^,^^36^

Ultimately, patient satisfaction levels were substantially significant in our study, followed by a considerably lower incidence of injection pain in the ciprofol group. Akhtar et al. (2024), in a meta-analysis of 1958 patients, registered a great lower incidence of injection pain during ciprofol administration in comparison with propofol for anesthesia induction.^37^ Still, a study measured patient satisfaction and injection pain by comparing two cohorts, one for patients under ciprofol and the other for people under propofol, and the results were higher satisfaction and significantly lower pain injection in the ciprofol group.^38^ The lower satisfaction of the anesthetic procedure might be related to the pain associated with propofol injections, due to its direct irritation of the venous endothelium, leading to the release of mediators like bradykinin, which increase vascular permeability and stimulate pain receptors. The reduced incidence of injection pain with ciprofol may be attributed to differences in its lipid emulsion formulation and pH, which could lead to less endothelial irritation compared to propofol.^6^^,^^39^ Improved patient comfort and satisfaction are also clinically relevant, as they may encourage adherence to colorectal cancer screening programs that require repeated colonoscopies, ultimately contributing to better public health outcomes.

Overall, these findings indicate that ciprofol may be a suitable alternative to propofol for colonoscopy sedation, offering comparable efficacy with a potentially more favorable safety and tolerability profile. Specifically, its lower incidence of respiratory depression, hypotension, and injection pain, alongside higher patient satisfaction, are encouraging. However, these differences should be interpreted cautiously due to the small evidence base and limited geographic scope of available studies. While the pharmacological properties of ciprofol suggest clinical advantages, further research is needed to determine whether these translate into meaningful improvements such as reduced post-procedural complications, shorter recovery times, or decreased hospital admissions*.* The rapid recovery and short post-procedure stay, which were assessed through standardized scoring systems such as the PADSS and modified Aldrete scores, are key clinical benefits that further support ciprofol's use for colonoscopy.

### Limitations

Despite providing valuable insights, this review has several limitations. First, the analysis included only three RCTs with relatively small sample sizes, which limits statistical power and increases susceptibility to heterogeneity. Second, all included studies were conducted in China, which may restrict the generalizability of our findings to broader and more diverse populations and healthcare systems. Third, the included patients were predominantly ASA PS I–II, restricting applicability to higher-risk populations. Fourth, the trials primarily assessed immediate procedural outcomes, while long-term safety and efficacy data remain unavailable. Fifth, potential publication bias and methodological heterogeneity (e.g., differences in propofol dosage and premedication protocols) cannot be excluded. Finally, only studies published in English were included, which may have introduced language bias. However, this decision was made to ensure accurate comprehension of the manuscripts and minimize the risk of misinterpretation during data extraction and analysis. Collectively, these limitations highlight the need for larger, multicenter, international RCTs to rigorously evaluate the short- and long-term safety, efficacy, and cost-effectiveness of ciprofol in colonoscopy.

## Conclusion

This meta-analysis provides the most comprehensive comparison to date between ciprofol and propofol in colonoscopy sedation. The available evidence suggests that ciprofol may offer comparable efficacy to propofol, with similar procedure success rates and sedation onset times(s). In addition, ciprofol was associated with a lower risk of respiratory depression, injection pain, and hypotension, while patient satisfaction appeared slightly higher. No significant differences were observed regarding bradycardia occurrence. Nevertheless, these findings should be interpreted with caution due to the limited number of studies and their geographic concentration, which restrict generalizability. Future high-quality, multicenter RCTs are warranted to confirm these results and to further assess long-term safety, cost-effectiveness, and the potential role of ciprofol as an alternative sedative and anesthetic in colonoscopy.

## Authors' contributions

Saul Dominici contributed to the conception and design of the study, data acquisition, drafting of the initial manuscript, critical revision, and supervision of the project.

Italo C. Martins contributed to data analysis, manuscript drafting, and critical revision.

Breno Dias L. Ribeiro contributed to data collection, manuscript drafting, and critical revision.

Victor Arthur Ohannesian contributed to data interpretation, data visualization, manuscript drafting, and critical revision.

Brunno Braga Sauaia contributed to data analysis, data visualization, manuscript drafting, and critical revision.

Abdias Rocha Santos contributed to study conception, project supervision, and critical revision.

Caio Márcio Barros de Oliveira contributed to project supervision, result validation, data interpretation, and critical revision.

Plinio da Cunha Leal contributed to project supervision, result validation, data interpretation, and critical revision.

All authors approved the final version of the manuscript and agree to be accountable for all aspects of the work, ensuring that any questions related to the accuracy or integrity of any part of the work are appropriately investigated and resolved.

## Funding

This research did not receive any specific grant from funding agencies in the public, commercial, or not-for-profit sectors.

## Data availability statement

No new data were created or analyzed in this study. Data sharing is not applicable to this article.

## Declaration of competing interest

The authors declare no conflicts of interest.

## Appendix Supplementary materials

Image, application 1

## Footnotes

Supplementary material associated with this article can be found in the online version at doi:10.1016/j.bjane.2025.844710.
