# Supplementary material for: Ciprofol versus propofol for sedation in colonoscopy: a systematic review and meta-analysis of randomized controlled trials
Source: Braz J Anesthesiol. 2025 Nov 17;76(1):844710. doi: 10.1016/j.bjane.2025.844710 (PMC12853187; doi:10.1016/j.bjane.2025.844710)
Supplement: Supplementary file 1 [file mmc1.docx]

**BJAN-D-25-00344_ Supplementary** **Material**

**Supplementary** **Material 1** Leave-one-out analysis of onset time to sedation(s).


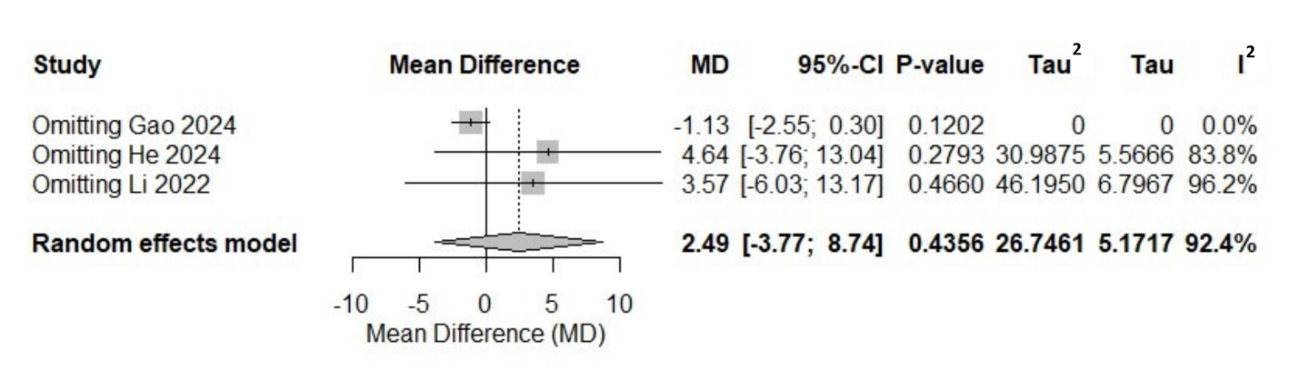


**Supplementary** **Material 2** Leave-one-out analysis of hypotension risk.


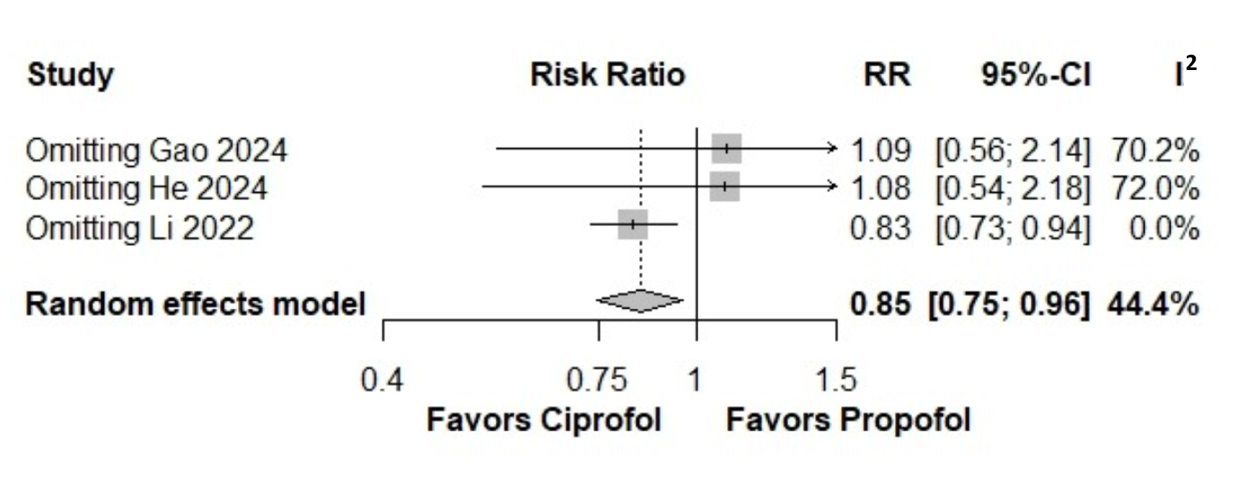


**Supplementary Material 3**

Revised Cochrane risk-of-bias tool for randomized trials (RoB 2)

TEMPLATE FOR COMPLETION

Edited by Julian PT Higgins, Jelena Savović, Matthew J Page, Jonathan AC Sterne
on behalf of the RoB2 Development Group

**Version of 22 August 2019**

The development of the RoB 2 tool was supported by the MRC Network of Hubs for Trials Methodology Research (MR/L004933/2- N61), with the support of the host MRC ConDuCT-II Hub (Collaboration and innovation for Difficult and Complex randomised controlled Trials In Invasive procedures - MR/K025643/1), by MRC research grant MR/M025209/1, and by a grant from The Cochrane Collaboration.


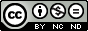


This work is licensed under a [Creative Commons Attribution-NonCommercial-NoDerivatives 4.0 International License](http://creativecommons.org/licenses/by-nc-nd/4.0/).

| **Study details**   \| **Reference** \| He KQ, Huang TT, Tan MY, Gao C, Wang S. Efficacy and Safety of Ciprofol Versus Propofol as Anesthetic for Patients Undergoing Painless Colonoscopy. Pain Ther. 2024 Dec;13(6):1633-1644. doi: 10.1007/s40122-024-00662-x. Epub 2024 Oct 14. PMID: 39400664; PMCID: PMC11543975. \| \| --- \| --- \|   **Study design**   \| X \| Individually-randomized parallel-group trial \| \| --- \| --- \| \| ⬜ \| Cluster-randomized parallel-group trial \| \| ⬜ \| Individually randomized cross-over (or other matched) trial \|   **For the purposes of this assessment, the interventions being compared are defined as**   \| Experimental: \| Ciprofol \| Comparator: \| Propofol \| \| --- \| --- \| --- \| --- \|  \| **Specify which outcome is being assessed for risk of bias** \| **Primary outcome:** Incidence of hypotension (SBP drop ≥ 20% from baseline) \| \| --- \| --- \|  \| **Specify the numerical result being assessed.** In case of multiple alternative analyses being presented, specify the numeric result (e.g. RR = 1.52 (95% CI 0.83 to 2.77) and/or a reference (e.g. to a table, figure or paragraph) that uniquely defines the result being assessed. \| Effect of assignment to intervention (intention-to-treat analysis) \| \| --- \| --- \|   **Is the review team’s aim for this result…?**   \| ⬜ \| to assess the effect of *assignment to intervention* (the ‘intention-to-treat’ effect) \| \| --- \| --- \| \| ⬜ \| to assess the effect of *adhering to intervention* (the ‘per-protocol’ effect) \|   **If the aim is to assess the effect of *adhering to intervention***, select the deviations from intended intervention that should be addressed (at least one must be checked):  ⬜ occurrence of non-protocol interventions  ⬜ failures in implementing the intervention that could have affected the outcome  ⬜ non-adherence to their assigned intervention by trial participants  **Which of the following sources were obtained to help inform the risk-of-bias assessment? (tick as many as apply)**  X Journal article(s) with results of the trial  ⬜ Trial protocol  ⬜ Statistical analysis plan (SAP)  X Non-commercial trial registry record (e.g. ClinicalTrials.gov record)  ⬜ Company-owned trial registry record (e.g. GSK Clinical Study Register record)  ⬜ “Grey literature” (e.g. unpublished thesis)  ⬜ Conference abstract(s) about the trial  ⬜ Regulatory document (e.g. Clinical Study Report, Drug Approval Package)  ⬜ Research ethics application  ⬜ Grant database summary (e.g. NIH RePORTER or Research Councils UK Gateway to Research)  ⬜ Personal communication with trialist  ⬜ Personal communication with the sponsor |
| --- | --- | --- | --- | --- | --- | --- | --- | --- | --- | --- | --- | --- | --- | --- | --- | --- | --- | --- | --- | --- |

## Risk of bias assessment

Responses underlined in green are potential markers for low risk of bias, and responses in red are potential markers for a risk of bias. Where questions relate only to sign posts to other questions, no formatting is used.

**Domain 1: Risk of bias arising from the randomization process**

| **Signalling questions** | **Comments** | **Response options** |
| --- | --- | --- |
| **1.1 Was the allocation sequence random?** |  | Y |
| **1.2 Was the allocation sequence concealed until participants were enrolled and assigned to interventions?** |  | Y |
| **1.3 Did baseline differences between intervention groups suggest a problem with the randomization process?** |  | N |
| **Risk-of-bias judgement** |  | Low |
| Optional: What is the predicted direction of bias arising from the randomization process? |  | NA |

Domain 2: Risk of bias due to deviations from the intended interventions (*effect of assignment to intervention*)

| **Signalling questions** | **Comments** | **Response options** |
| --- | --- | --- |
| **2.1. Were participants aware of their assigned intervention during the trial?** | (double-blind design) **(blinded administration and identical syringes)** | N |
| **2.2. Were carers and people delivering the interventions aware of participants' assigned intervention during the trial?** |  | N |
| **2.3. If Y/PY/NI to 2.1 or 2.2: Were there deviations from the intended intervention that arose because of the trial context?** |  | N |
| **2.4 If Y/PY to 2.3: Were these deviations likely to have affected the outcome?** |  | N |
| **2.5. If Y/PY/NI to 2.4: Were these deviations from intended intervention balanced between groups?** |  | Y |
| **2.6 Was an appropriate analysis used to estimate the effect of assignment to intervention?** |  | Y |
| **2.7 If N/PN/NI to 2.6: Was there potential for a substantial impact (on the result) of the failure to analyse participants in the group to which they were randomized?** |  | N |
| **Risk-of-bias judgement** |  | Low |
| Optional: What is the predicted direction of bias due to deviations from intended interventions? |  | NA |

Domain 3: Missing outcome data

| **Signalling questions** | **Comments** | **Response options** |
| --- | --- | --- |
| **3.1 Were data for this outcome available for all, or nearly all, participants randomized?** | (No significant missing data reported) | Y |
| **3.2 If N/PN/NI to 3.1: Is there evidence that the result was not biased by missing outcome data?** | X | X |
| **3.3 If N/PN to 3.2: Could missingness in the outcome depend on its true value?** | X | X |
| **3.4 If Y/PY/NI to 3.3: Is it likely that missingness in the outcome depended on its true value?** |  | X |
| **Risk-of-bias judgement** |  | Low |
| Optional: What is the predicted direction of bias due to missing outcome data? |  | NA |

Domain 4: Risk of bias in measurement of the outcome

| **Signalling questions** | **Comments** | **Response options** |
| --- | --- | --- |
| **4.1 Was the method of measuring the outcome inappropriate?** | (BP measured objectively with standard equipment) | N |
| **4.2 Could measurement or ascertainment of the outcome have differed between intervention groups?** | (Standardized BP measurement protocol used) | N |
| **4.3 If N/PN/NI to 4.1 and 4.2: Were outcome assessors aware of the intervention received by study participants?** | (Blinded assessment) | N |
| **4.4 If Y/PY/NI to 4.3: Could assessment of the outcome have been influenced by knowledge of intervention received?** |  | N |
| **4.5 If Y/PY/NI to 4.4: Is it likely that assessment of the outcome was influenced by knowledge of intervention received?** |  | N |
| **Risk-of-bias judgement** |  | Low |
| Optional: What is the predicted direction of bias in measurement of the outcome? |  | NA / Favours experimental / Favours comparator / Towards null /Away from null / Unpredictable |

Domain 5: Risk of bias in selection of the reported result

| **Signalling questions** | **Comments** | **Response options** |
| --- | --- | --- |
| **5.1 Were the data that produced this result analysed in accordance with a pre-specified analysis plan that was finalized before unblinded outcome data were available for analysis?** |  | Y |
| **Is the numerical result being assessed likely to have been selected, on the basis of the results, from...** |  |  |
| **5.2. ... multiple eligible outcome measurements (e.g. scales, definitions, time points) within the outcome domain?** |  | N |
| **5.3 ... multiple eligible analyses of the data?** |  | N |
| **Risk-of-bias judgement** |  | Low |
| Optional: What is the predicted direction of bias due to selection of the reported result? |  | NA |

Overall risk of bias

| **Risk-of-bias judgement** |  | Low |
| --- | --- | --- |
| Optional: What is the overall predicted direction of bias for this outcome? |  | NA |


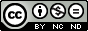


This work is licensed under a [Creative Commons Attribution-NonCommercial-NoDerivatives 4.0 International License](http://creativecommons.org/licenses/by-nc-nd/4.0/).

Revised Cochrane risk-of-bias tool for randomized trials (RoB 2)

TEMPLATE FOR COMPLETION

Edited by Julian PT Higgins, Jelena Savović, Matthew J Page, Jonathan AC Sterne
on behalf of the RoB2 Development Group

**Version of 22 August 2019**

The development of the RoB 2 tool was supported by the MRC Network of Hubs for Trials Methodology Research (MR/L004933/2- N61), with the support of the host MRC ConDuCT-II Hub (Collaboration and innovation for Difficult and Complex randomised controlled Trials In Invasive procedures - MR/K025643/1), by MRC research grant MR/M025209/1, and by a grant from The Cochrane Collaboration.


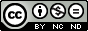


This work is licensed under a [Creative Commons Attribution-NonCommercial-NoDerivatives 4.0 International License](http://creativecommons.org/licenses/by-nc-nd/4.0/).

| **Study details**   \| **Reference** \| Li J, Wang X, Liu J, Wang X, Li X, Wang Y, Ouyang W, Li J, Yao S, Zhu Z, Guo Q, Yu Y, Meng J, Zuo Y. Comparison of ciprofol (HSK3486) versus propofol for the induction of deep sedation during gastroscopy and colonoscopy procedures: A multi-centre, non-inferiority, randomized, controlled phase 3 clinical trial. Basic Clin Pharmacol Toxicol. 2022 Aug;131(2):138-148. doi: 10.1111/bcpt.13761. Epub 2022 Jun 10. PMID: 35653554; PMCID: PMC9543620. \| \| --- \| --- \|   **Study design**   \| X \| Individually-randomized parallel-group trial \| \| --- \| --- \| \| ⬜ \| Cluster-randomized parallel-group trial \| \| ⬜ \| Individually randomized cross-over (or other matched) trial \|   **For the purposes of this assessment, the interventions being compared are defined as**   \| Experimental: \| Ciprofol \| Comparator: \| Propofol \| \| --- \| --- \| --- \| --- \|  \| **Specify which outcome is being assessed for risk of bias** \| Success rate of colonoscopy (completion without need for rescue sedation) \| \| --- \| --- \|  \| **Specify the numerical result being assessed.** In case of multiple alternative analyses being presented, specify the numeric result (e.g. RR = 1.52 (95% CI 0.83 to 2.77) and/or a reference (e.g. to a table, figure or paragraph) that uniquely defines the result being assessed. \| Effect of assignment to intervention (intention-to-treat analysis) \| \| --- \| --- \|   **Is the review team’s aim for this result…?**   \| ⬜ \| to assess the effect of *assignment to intervention* (the ‘intention-to-treat’ effect) \| \| --- \| --- \| \| ⬜ \| to assess the effect of *adhering to intervention* (the ‘per-protocol’ effect) \|   **If the aim is to assess the effect of *adhering to intervention***, select the deviations from intended intervention that should be addressed (at least one must be checked):  ⬜ occurrence of non-protocol interventions  ⬜ failures in implementing the intervention that could have affected the outcome  ⬜ non-adherence to their assigned intervention by trial participants  **Which of the following sources were obtained to help inform the risk-of-bias assessment? (tick as many as apply)**  X Journal article(s) with results of the trial  ⬜ Trial protocol  ⬜ Statistical analysis plan (SAP)  X Non-commercial trial registry record (e.g. ClinicalTrials.gov record)  ⬜ Company-owned trial registry record (e.g. GSK Clinical Study Register record)  ⬜ “Grey literature” (e.g. unpublished thesis)  ⬜ Conference abstract(s) about the trial  ⬜ Regulatory document (e.g. Clinical Study Report, Drug Approval Package)  ⬜ Research ethics application  ⬜ Grant database summary (e.g. NIH RePORTER or Research Councils UK Gateway to Research)  ⬜ Personal communication with trialist  ⬜ Personal communication with the sponsor |
| --- | --- | --- | --- | --- | --- | --- | --- | --- | --- | --- | --- | --- | --- | --- | --- | --- | --- | --- | --- | --- |

## Risk of bias assessment

Responses underlined in green are potential markers for low risk of bias, and responses in red are potential markers for a risk of bias. Where questions relate only to sign posts to other questions, no formatting is used.

**Domain 1: Risk of bias arising from the randomization process**

| **Signalling questions** | **Comments** | **Response options** |
| --- | --- | --- |
| **1.1 Was the allocation sequence random?** | (1:1 randomization using an interactive web response system - IWRS) (centralized randomization system ensured concealment) | Y |
| **1.2 Was the allocation sequence concealed until participants were enrolled and assigned to interventions?** |  | Y |
| **1.3 Did baseline differences between intervention groups suggest a problem with the randomization process?** | (balanced baseline characteristics between groups) | N |
| **Risk-of-bias judgement** |  | Low |
| Optional: What is the predicted direction of bias arising from the randomization process? |  | NA |

Domain 2: Risk of bias due to deviations from the intended interventions (*effect of assignment to intervention*)

| **Signalling questions** | **Comments** | **Response options** |
| --- | --- | --- |
| **2.1. Were participants aware of their assigned intervention during the trial?** | (double-blind design, patients blinded) (identical drug appearance, syringes covered, assessors blinded) | N |
| **2.2. Were carers and people delivering the interventions aware of participants' assigned intervention during the trial?** |  | N |
| **2.3. If Y/PY/NI to 2.1 or 2.2: Were there deviations from the intended intervention that arose because of the trial context?** | (strict protocol for sedation and rescue use of propofol pre-defined) | N |
| **2.4 If Y/PY to 2.3: Were these deviations likely to have affected the outcome?** |  | N |
| **2.5. If Y/PY/NI to 2.4: Were these deviations from intended intervention balanced between groups?** |  | Y |
| **2.6 Was an appropriate analysis used to estimate the effect of assignment to intervention?** | (ITT analysis performed) | Y |
| **2.7 If N/PN/NI to 2.6: Was there potential for a substantial impact (on the result) of the failure to analyse participants in the group to which they were randomized?** |  | N |
| **Risk-of-bias judgement** |  | Low |
| Optional: What is the predicted direction of bias due to deviations from intended interventions? |  | NA |

Domain 3: Missing outcome data

| **Signalling questions** | **Comments** | **Response options** |
| --- | --- | --- |
| **3.1 Were data for this outcome available for**  **all, or nearly all, participants randomized?** | (No major missing data reported, full follow-up achieved) | Y |
| **3.2 If N/PN/NI to 3.1: Is there evidence that the result was not biased by missing outcome data?** | X | X |
| **3.3 If N/PN to 3.2: Could missingness in the outcome depend on its true value?** | X | X |
| **3.4 If Y/PY/NI to 3.3: Is it likely that missingness in the outcome depended on its true value?** |  | X |
| **Risk-of-bias judgement** |  | Low |
| Optional: What is the predicted direction of bias due to missing outcome data? |  | NA |

Domain 4: Risk of bias in measurement of the outcome

| **Signalling questions** | **Comments** | **Response options** |
| --- | --- | --- |
| **4.1 Was the method of measuring the outcome inappropriate?** | (Objective sedation criteria and endoscopy success metrics used) | N |
| **4.2 Could measurement or ascertainment of the outcome have differed between intervention groups?** | (Blinded assessors, standardized assessment criteria used) | N |
| **4.3 If N/PN/NI to 4.1 and 4.2: Were outcome assessors aware of the intervention received by study participants?** | (Blinded evaluation ensured) | N |
| **4.4 If Y/PY/NI to 4.3: Could assessment of the outcome have been influenced by knowledge of intervention received?** |  | N |
| **4.5 If Y/PY/NI to 4.4: Is it likely that assessment of the outcome was influenced by knowledge of intervention received?** |  | N |
| **Risk-of-bias judgement** |  | Low |
| Optional: What is the predicted direction of bias in measurement of the outcome? |  | NA / Favours experimental / Favours comparator / Towards null /Away from null / Unpredictable |

Domain 5: Risk of bias in selection of the reported result

| **Signalling questions** | **Comments** | **Response options** |
| --- | --- | --- |
| **5.1 Were the data that produced this result analysed in accordance with a pre-specified analysis plan that was finalized before unblinded outcome data were available for analysis?** |  | Y |
| **Is the numerical result being assessed likely to have been selected, on the basis of the results, from...** |  |  |
| **5.2. ... multiple eligible outcome measurements (e.g. scales, definitions, time points) within the outcome domain?** |  | N |
| **5.3 ... multiple eligible analyses of the data?** |  | N |
| **Risk-of-bias judgement** |  | Low |
| Optional: What is the predicted direction of bias due to selection of the reported result? |  | NA |

Overall risk of bias

| **Risk-of-bias judgement** |  | Low |
| --- | --- | --- |
| Optional: What is the overall predicted direction of bias for this outcome? |  | NA |


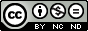


This work is licensed under a [Creative Commons Attribution-NonCommercial-NoDerivatives 4.0 International License](http://creativecommons.org/licenses/by-nc-nd/4.0/).

Revised Cochrane risk-of-bias tool for randomized trials (RoB 2)

TEMPLATE FOR COMPLETION

Edited by Julian PT Higgins, Jelena Savović, Matthew J Page, Jonathan AC Sterne
on behalf of the RoB2 Development Group

**Version of 22 August 2019**

The development of the RoB 2 tool was supported by the MRC Network of Hubs for Trials Methodology Research (MR/L004933/2- N61), with the support of the host MRC ConDuCT-II Hub (Collaboration and innovation for Difficult and Complex randomised controlled Trials In Invasive procedures - MR/K025643/1), by MRC research grant MR/M025209/1, and by a grant from The Cochrane Collaboration.


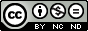


This work is licensed under a [Creative Commons Attribution-NonCommercial-NoDerivatives 4.0 International License](http://creativecommons.org/licenses/by-nc-nd/4.0/).

| **Study details**   \| **Reference** \| Gao SH, Tang QQ, Wang CM, Guan ZY, Wang LL, Zhang J, Yan ZL. The efficacy and safety of ciprofol and propofol in patients undergoing colonoscopy: A double-blind, randomized, controlled trial. J Clin Anesth. 2024 Aug;95:111474. doi: 10.1016/j.jclinane.2024.111474. Epub 2024 Apr 11. PMID: 38608531. \| \| --- \| --- \|   **Study design**   \| X \| Individually-randomized parallel-group trial \| \| --- \| --- \| \| ⬜ \| Cluster-randomized parallel-group trial \| \| ⬜ \| Individually randomized cross-over (or other matched) trial \|   **For the purposes of this assessment, the interventions being compared are defined as**   \| Experimental: \| Ciprofol \| Comparator: \| Propofol \| \| --- \| --- \| --- \| --- \|  \| **Specify which outcome is being assessed for risk of bias** \| **Success rate of colonoscopy (completion without need for rescue sedation)** \| \| --- \| --- \|  \| **Specify the numerical result being assessed.** In case of multiple alternative analyses being presented, specify the numeric result (e.g. RR = 1.52 (95% CI 0.83 to 2.77) and/or a reference (e.g. to a table, figure or paragraph) that uniquely defines the result being assessed. \| Effect of assignment to intervention (intention-to-treat analysis) \| \| --- \| --- \|   **Is the review team’s aim for this result…?**   \| ⬜ \| to assess the effect of *assignment to intervention* (the ‘intention-to-treat’ effect) \| \| --- \| --- \| \| ⬜ \| to assess the effect of *adhering to intervention* (the ‘per-protocol’ effect) \|   **If the aim is to assess the effect of *adhering to intervention***, select the deviations from intended intervention that should be addressed (at least one must be checked):  ⬜ occurrence of non-protocol interventions  ⬜ failures in implementing the intervention that could have affected the outcome  ⬜ non-adherence to their assigned intervention by trial participants  **Which of the following sources were obtained to help inform the risk-of-bias assessment? (tick as many as apply)**  X Journal article(s) with results of the trial  ⬜ Trial protocol  ⬜ Statistical analysis plan (SAP)  X Non-commercial trial registry record (e.g. ClinicalTrials.gov record)  ⬜ Company-owned trial registry record (e.g. GSK Clinical Study Register record)  ⬜ “Grey literature” (e.g. unpublished thesis)  ⬜ Conference abstract(s) about the trial  ⬜ Regulatory document (e.g. Clinical Study Report, Drug Approval Package)  ⬜ Research ethics application  ⬜ Grant database summary (e.g. NIH RePORTER or Research Councils UK Gateway to Research)  ⬜ Personal communication with trialist  ⬜ Personal communication with the sponsor |
| --- | --- | --- | --- | --- | --- | --- | --- | --- | --- | --- | --- | --- | --- | --- | --- | --- | --- | --- | --- | --- |

## Risk of bias assessment

Responses underlined in green are potential markers for low risk of bias, and responses in red are potential markers for a risk of bias. Where questions relate only to sign posts to other questions, no formatting is used.

**Domain 1: Risk of bias arising from the randomization process**

| **Signalling questions** | **Comments** | **Response options** |
| --- | --- | --- |
| **1.1 Was the allocation sequence random?** | (computer-generated randomization) (allocation concealed by an independent assistant) | Y |
| **1.2 Was the allocation sequence concealed until participants were enrolled and assigned to interventions?** |  | Y |
| **1.3 Did baseline differences between intervention groups suggest a problem with the randomization process?** | (balanced demographics and baseline vitals reported) | N |
| **Risk-of-bias judgement** |  | Low |
| Optional: What is the predicted direction of bias arising from the randomization process? |  | NA |

Domain 2: Risk of bias due to deviations from the intended interventions (*effect of assignment to intervention*)

| **Signalling questions** | **Comments** | **Response options** |
| --- | --- | --- |
| **2.1. Were participants aware of their assigned intervention during the trial?** | (double-blind design) **(identical drug appearance and blinding ensured)** | N |
| **2.2. Were carers and people delivering the interventions aware of participants' assigned intervention during the trial?** |  | N |
| **2.3. If Y/PY/NI to 2.1 or 2.2: Were there deviations from the intended intervention that arose because of the trial context?** | (strict protocol for sedation and rescue use of propofol pre-defined) | N |
| **2.4 If Y/PY to 2.3: Were these deviations likely to have affected the outcome?** |  | N |
| **2.5. If Y/PY/NI to 2.4: Were these deviations from intended intervention balanced between groups?** |  | Y |
| **2.6 Was an appropriate analysis used to estimate the effect of assignment to intervention?** | (ITT analysis performed, predefined protocol for alternative sedation use) | Y |
| **2.7 If N/PN/NI to 2.6: Was there potential for a substantial impact (on the result) of the failure to analyse participants in the group to which they were randomized?** |  | N |
| **Risk-of-bias judgement** |  | Low |
| Optional: What is the predicted direction of bias due to deviations from intended interventions? |  | NA |

Domain 3: Missing outcome data

| **Signalling questions** | **Comments** | **Response options** |
| --- | --- | --- |
| **3.1 Were data for this outcome available for all, or nearly all, participants randomized?** | (No major missing data reported, full follow-up achieved) | Y |
| **3.2 If N/PN/NI to 3.1: Is there evidence that the result was not biased by missing outcome data?** | X | X |
| **3.3 If N/PN to 3.2: Could missingness in the outcome depend on its true value?** | X | X |
| **3.4 If Y/PY/NI to 3.3: Is it likely that missingness in the outcome depended on its true value?** |  | X |
| **Risk-of-bias judgement** |  | Low |
| Optional: What is the predicted direction of bias due to missing outcome data? |  | NA |

Domain 4: Risk of bias in measurement of the outcome

| **Signalling questions** | **Comments** | **Response options** |
| --- | --- | --- |
| **4.1 Was the method of measuring the outcome inappropriate?** | (Objective sedation criteria and endoscopy success metrics used) | N |
| **4.2 Could measurement or ascertainment of the outcome have differed between intervention groups?** | (Blinded outcome assessors and standardized assessment tools used) | N |
| **4.3 If N/PN/NI to 4.1 and 4.2: Were outcome assessors aware of the intervention received by study participants?** | (Blinded anesthesiologist responsible for data collection) | N |
| **4.4 If Y/PY/NI to 4.3: Could assessment of the outcome have been influenced by knowledge of intervention received?** |  | N |
| **4.5 If Y/PY/NI to 4.4: Is it likely that assessment of the outcome was influenced by knowledge of intervention received?** |  | N |
| **Risk-of-bias judgement** |  | Low |
| Optional: What is the predicted direction of bias in measurement of the outcome? |  | NA / Favours experimental / Favours comparator / Towards null /Away from null / Unpredictable |

Domain 5: Risk of bias in selection of the reported result

| **Signalling questions** | **Comments** | **Response options** |
| --- | --- | --- |
| **5.1 Were the data that produced this result analysed in accordance with a pre-specified analysis plan that was finalized before unblinded outcome data were available for analysis?** |  | Y |
| **Is the numerical result being assessed likely to have been selected, on the basis of the results, from...** |  |  |
| **5.2. ... multiple eligible outcome measurements (e.g. scales, definitions, time points) within the outcome domain?** |  | N |
| **5.3 ... multiple eligible analyses of the data?** |  | N |
| **Risk-of-bias judgement** |  | Low |
| Optional: What is the predicted direction of bias due to selection of the reported result? |  | NA |

Overall risk of bias

| **Risk-of-bias judgement** |  | Low |
| --- | --- | --- |
| Optional: What is the overall predicted direction of bias for this outcome? |  | NA |


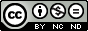


This work is licensed under a [Creative Commons Attribution-NonCommercial-NoDerivatives 4.0 International License](http://creativecommons.org/licenses/by-nc-nd/4.0/).
